# Multifactorial discrimination as a fundamental cause of mental health inequities

**DOI:** 10.1186/s12939-017-0532-z

**Published:** 2017-03-04

**Authors:** Mariam Khan, Misja Ilcisin, Katherine Saxton

**Affiliations:** 1 Public Health Program, Santa Clara University, 500 El Camino Real, Santa Clara, CA 95053 USA; 2 Department of Biology, Santa Clara University, 500 El Camino Real, Santa Clara, CA 95053 USA

**Keywords:** Health equity, Fundamental causes, Discrimination, Intersectionality, Mental Health

## Abstract

**Background:**

The theory of fundamental causes explains why health disparities persist over time, even as risk factors, mechanisms, and diseases change. Using an intersectional framework, we evaluated multifactorial discrimination as a fundamental cause of mental health disparities.

**Methods:**

Using baseline data from the *Project STRIDE: Stress, Identity, and Mental Health* study, we examined the health effects of discrimination among individuals who self-identified as lesbian, gay, or bisexual. We used logistic and linear regression to assess whether multifactorial discrimination met the four criteria designating a fundamental cause, namely that the cause: 1) influences multiple health outcomes, 2) affects multiple risk factors, 3) involves access to resources that can be leveraged to reduce consequences of disease, and 4) reproduces itself in varied contexts through changing mechanisms.

**Results:**

Multifactorial discrimination predicted high depression scores, psychological well-being, and substance use disorder diagnosis. Discrimination was positively associated with risk factors for high depression scores: chronic strain and total number of stressful life events. Discrimination was associated with significantly lower levels of mastery and self-esteem, protective factors for depressive symptomatology. Even after controlling for risk factors, discrimination remained a significant predictor for high depression scores. Among subjects with low depression scores, multifactorial discrimination also predicted anxiety and aggregate mental health scores.

**Conclusions:**

Multifactorial discrimination should be considered a fundamental cause of mental health inequities and may be an important cause of broad health disparities among populations with intersecting social identities.

## Background

To address the challenges of health inequities and the pervasive consequences of the social determinants of health, continued attention must be paid to the health impacts of social marginalization [1]. Health disparities based on sexual orientation, gender, race, socioeconomic status (SES), and other identity markers may be viewed through the minority stress framework [2], which suggests that unique stressors arising from minority experiences can impact epigenetic processes [3], influence health behaviors [4], and modulate the use of health services [5]. While minority stress may, in part, account for disparities due to the stressful experiences of discrimination [6, 7], the persisting and pervasive nature of these disparities can best be explained by the theory of fundamental causes [8]. According to the fundamental cause theory, despite radical changes in disease mechanisms and risk factors over time, health inequalities persist and replicate, such that health status and outcomes consistently differ across population groups. These disparities are reproduced via the ability of advantaged groups to access more resources-including knowledge, political clout, finances, and social support-than disadvantaged groups.

We expand on recent research showing that racism may be a fundamental cause of health inequalities [9] by examining the associations between intersecting minority identities, the stress experienced by non-White sexual minorities, and mental health outcomes. Given their dual minority status, non-White sexual minorities may experience stressors that differ from those experienced by non-White heterosexuals or White lesbian, gay, bisexual, and queer individuals (LGBQs). Within LGBQ communities, non-White sexual minorities often experience racism that causes them to feel excluded from LGBQ community spaces and events [10]. For example, certain gay bars have been known to provide poor services to African American gay men and in some cases, refuse their entry altogether [11]. Within their own cultural communities, non-White sexual minorities may experience heterosexism causing members to conceal their sexual orientation [12]. Such forms of discrimination can have major implications for health, leading to poor mental and physical health outcomes among non-White sexual minorities [13, 14].

To investigate the effects of multifactorial discrimination on mental health outcomes among multiple minority populations, we incorporated an intersectional framework into our understanding of discrimination as a potential fundamental cause of mental health inequalities. Intersectionality is a framework used to conceptualize how multiple social identities dynamically intersect and concurrently interact with each other in relation to interlocking structures of oppression [15]. Intersectionality allows for a nuanced understanding of how intersecting identities confer unique privileges and oppressions. As a consequence of an individual’s intersecting identities, the oppressions they experience are intrinsically linked and cannot be isolated from another. Within an intersectional framework, it is difficult to examine different types of discrimination independently, because they constitutively overlap and interact. An intersectional approach to discrimination recognizes that discrimination is multifactorial, arising from diverse sources and for numerous reasons, impacting multiple minority identities. Multifactorial discrimination refers to the total discrimination experienced by such individuals, accounting for their sexual orientation, gender, race/ethnicity, and/or other identity markers.

Due to the importance of discrimination in understanding the health of multiple minority identities, we sought to test whether experiences of multifactorial discrimination can be identified as a fundamental cause of mental health inequalities. In order to test this theory, we compared multifactorial discrimination to Link and Phelan’s four necessary criteria to establish a fundamental cause: 1) the cause influences multiple health outcomes; 2) the cause affects health outcomes through multiple risk factors; 3) the cause involves access to resources that can be used to reduce health risks or the progression of disease; and 4) the association between the cause and poor health should be reproduced over time through varied mechanisms [8]. As a result of the limitations of our data, we chose to focus this study specifically on evaluating multifactorial discrimination as a fundamental cause of mental health inequalities among lesbian, gay, and bisexual (LGB) populations.

## Methods

### Sample

We conducted our analysis using publicly available data from *Project STRIDE: Stress, Identity, and Mental Health*, accessed via the Interuniversity Consortium for Political and Social Research (University of Michigan) [16]. In our analysis, we exclusively used the baseline data from *Project STRIDE. Project STRIDE* is a mixed-methods study funded by the National Institute of Mental Health, which examined the associations between minority identities and stress, health outcomes, coping abilities, and social support resources. The total baseline sample consisted of 524 participants who self-identified as straight, lesbian, gay, or bisexual (LGB). Study participants were selected such that all heterosexual subjects in the sample identified as White (i.e., the sample did not include Black/African-American or Latino/Hispanic participants who identified as heterosexual). Therefore, we excluded heterosexual subjects from our analysis and used only the LGB sample (*N* = 396). In the LGB sample, 50.13% identified as male and 49.87% as female. 32.91% of the subjects identified as Black/African-American, 33.16% as Latino/Hispanic, and 33.92% as White. Detailed information describing *Project STRIDE’s* sampling methodology is available online [17].

### Measures

#### Sociodemographic variables

Education level, nativity status, age, employment status, sex at birth, race/ethnicity, and income were described for the sample. Income quintiles were approximated using the New York state quintiles listed by Assets & Opportunity Scorecard [18].

#### Multifactorial discrimination

In the *Project STRIDE* study, discrimination was measured via a modified version of The Everyday Discrimination Scale, which is routinely used to assess experiences of unfair treatment [19]. The scale was adapted to apply to all minority groups in the study.

#### Depression

We chose depression as our primary health outcome of interest. Depression scores were calculated using the Center for Epidemiological Studies depression scale (CESD; 20-items, alpha = 0.92), which measured depressive symptoms experienced over the week prior to the subject’s interview [20]. Scores ranged from 0 to 60; scores of 16 and above were categorized as high depression scores.

#### Additional mental health outcomes

In addition to high depression scores, we examined psychological well-being (18-items, alpha = .25–.55), aggregate mental health scores, and formal diagnoses of anxiety and substance use disorders. The psychological well-being scale focused on dimensions that included self-acceptance, purpose in life, and personal growth [21, 22]. Anxiety and substance use disorders were assessed using the World Mental Health Composite International Diagnostic Interview, a structured diagnostic interview [23]. Aggregate mental health was assessed via the 12-item Short Form survey [24].

#### Protective factors

Mastery (7-items, alpha = 0.64), Self Esteem (10-items, alpha = 0.86), Collective Esteem (16-items, alpha = 0.70 -0.80), and Social Support Network size were all considered possible protective factors for high depression scores. Mastery measured the extent to which subjects felt they had control over certain aspects of their lives [25]. The Self Esteem scale assessed both positive and negative self-perception [26]. The Collective Esteem scale measured subjects’ evaluation of their group memberships [27]. Social Network Size captured the number of people who provided support to the subject over the previous year [28].

### Stressful life events

Stressful Life Events was one of the two primary risk factors examined in this paper. The Stressful Life Events questionnaire was adapted from the Structured Event Probe and Narrative Rating scale and measured stressful experiences through the assessment of 43 possible stressful life events [29, 30].

### Chronic strain

The chronic strain assessment measured long term strain as on-going sources of strain in nine possible areas life, including relationships, financial strain, work, and general life problems [31]. Higher scores reflect a higher level of chronic strain. Chronic strain was one of the two primary risk factors examined in this paper. The correlation between chronic strain and stressful life events in this sample was small to moderate (r = 0.27, *p* < 0.01).

### Stigma

A stigma questionnaire, generalized to measure stigma across multiple statuses and characteristics, was used (6-items, alpha = 0.88) [28, 32]*.* The final scale captured stigma resulting from gender, race, sexual orientation, nationality, ethnicity, and socioeconomic status.

### Statistical analysis

Differences in sociodemographic and health characteristics between participants with high and low depression scores were examined via $\chi$ ^2^ tests. We used both linear and logistic regression models to test multifactorial discrimination against all four fundamental cause criteria, as described below. All regression models were limited to LGB participants and adjusted for race/ethnicity and sex at birth. Statistical significance was defined as *p* < 0.05. All analysis was conducted using Stata 12 (Stata Corp LP, College Station, TX).

### Evaluation of the four fundamental cause criteria

1. We first investigated whether multifactorial discrimination was associated with mental health outcomes. We used logistic regression models to test the associations between discrimination and high depression scores as well as between discrimination and substance use disorder. We used linear regression to examine the association between discrimination and psychological well-being. All models were adjusted for race/ethnicity and sex. Results of criterion 1 analysis are shown in Table 2.
2. To evaluate whether discrimination affected multiple risk factors of depression, we first determined that chronic strain and total stressful life events were positively associated with high depression scores. We then used linear regression to assess whether discrimination was a significant predictor of chronic strain and stressful life events, adjusting for race/ethnicity and sex. Results of criterion 2 analysis are shown in Table 3.
3. To test whether discrimination predicted access to psychosocial protective factors for depression, we first used linear regression models to evaluate whether mastery, self-esteem, collective esteem, and social support network size were negatively associated with depression. We then used linear regression to determine if discrimination was inversely associated with these protective factors. All models were adjusted for race/ethnicity and sex. Results of criterion 3 analysis are shown in Table 4.
4. In order to evaluate whether discrimination can affect mental health through the replacement of intervening mechanisms, we conducted two analyses. We first used logistic regression to determine whether discrimination predicted high depression scores, even after controlling for risk factors for depression (i.e., chronic strain and stressful life events). This analysis allowed us to evaluate whether the relationship between discrimination and depression was mediated by chronic strain and stressful life events, or whether additional, unmeasured mechanisms also influenced depression.
  Next, to examine the relationship between discrimination and mental health outcomes other than depression, we used linear regression to assess the relationship between discrimination and aggregate mental health score and logistic regression to assess the relationship between discrimination and anxiety disorder diagnoses among individuals with low depression scores. This analysis allowed us to test if multifactorial discrimination played a significant role in the mental health among individuals who did not have high depressive symptoms. All models were adjusted for race/ethnicity and sex. Results of criterion 4 analysis are shown in Table 5.

### Sensitivity analysis

In the *Project STRIDE* dataset, the variables for discrimination and stigma were moderately correlated (r = 0.54, *p* < 0.01). To ensure that our analysis identified multifactorial discrimination as a fundamental cause separate from stigma, a factor that has been previously identified as a fundamental cause of health inequalities [33], we repeated all four steps of our analysis twice: first by using stigma in place of discrimination, and then by including both discrimination and stigma in the regression models.

## Results

Table 1 presents the demographics and health characteristics of LGB individuals with low and high depression scores. Lower education levels were associated with an increased probability of depression ($\chi$ ^2^ = 13.79, *p* < 0.05). Among individuals with high levels of depression, 42.17% had completed a bachelor’s degree or more advanced education, yet over 72.92% belonged to the lowest two income quintiles ($\chi$ ^2^ = 10.62, *p* < 0.05). Nearly half of Latino/Hispanic participants (49.62%) reported high depression symptoms, higher than the risk of depression among White (32.09%) or Black/African-American (30.00%) participants ($\chi$ ^2^ = 13.03, *p* < 0.01). People who were unemployed ($\chi$ ^2^ = 6.14, *p* < 0.05) or diagnosed with an emotional disorder ($\chi$ ^2^ = 14.32, *p* < 0.01) were more likely to report high depression symptoms.

**Table 1:**
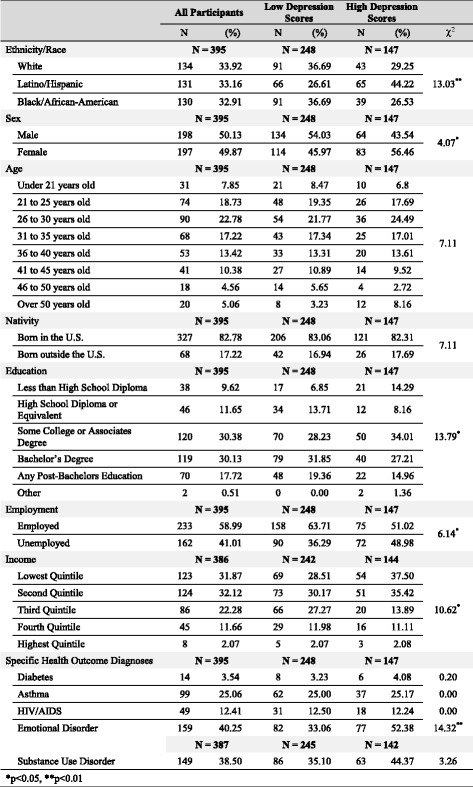
Demographics and health characteristics of LGB participants with high and low depression scores

### Criterion 1: multifactorial discrimination predicts health outcomes

Multifactorial discrimination significantly predicted multiple health outcomes, adjusting for race/ethnicity and sex. Higher levels of discrimination were associated with increased odds of depression (Adj. OR = 2.23; 95% CI = 1.54–3.23), reduced psychological well-being (Adj. β = −0.30; 95% CI = −0.42–-0.18), and increased odds of a substance use disorder diagnosis (Adj. OR = 2.12; 95% CI = 1.47–3.06), as seen in Table 2.

**Table 2:**
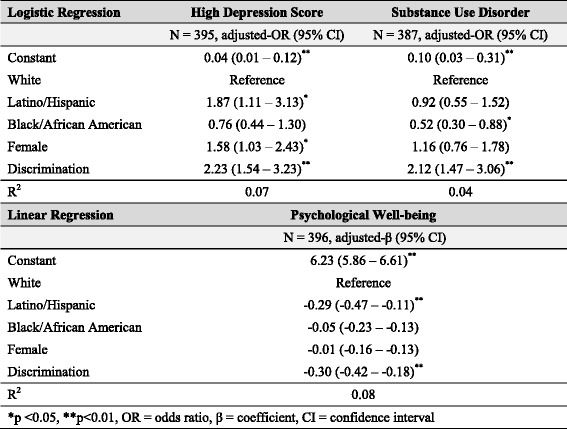
Effect of discrimination on health outcomes

Patterns of poor health differed by sex and race/ethnicity (Table 2). Women were more likely to have high depression scores than men (Adj. OR = 1.58; 95% CI = 1.03–2.43). Latino/Hispanics were more likely to have high depression scores (Adj. OR = 1.87; 95% CI = 1.11–3.13) and lower psychological well-being (Adj. β = −0.29; 95% CI = −0.47 – -0.11), while Black/African-Americans were less likely to be diagnosed with a substance use disorder (Adj. OR = 0.52; 95% CI = 0.30–0.88) than Whites. These different health patterns according to biological sex and race/ethnicity among LGBs allude to the unique experiences of each group and reinforce the importance of an intersectional framework for addressing health inequalities.

### Criterion 2: multifactorial discrimination predicts risk factors for high depression

Chronic strain and stressful life events were each risk factors for depression, and both were associated with multifactorial discrimination. Adjusting for race/ethnicity and sex, chronic strain was associated with increased odds of depression (Adj. OR = 32.30; 95% CI = 10.70–97.52). Adjusting for race/ethnicity and sex, stressful life events were positively associated with increased odds of depression (Adj. OR = 1.04; 95% CI = 1.00–1.08).

Compared to White men, women (Adj. β = 0.05; 95% CI = 0.01–0.09), Black/African-Americans (Adj. β = 0.07; 95% CI = 0.02–0.12), and Latino/Hispanics (Adj. β = 0.12; 95% CI = 0.07–0.17) experienced higher levels of chronic strain. Both Black/African-Americans (Adj. β = 1.53; 95% CI = 0.27–2.79) and Latino/Hispanics (Adj. β = 1.55; 0.29–2.81) experienced higher numbers of stressful life events than did White participants.

Increased experiences of discrimination were associated with both increased chronic strain (Adj. β = 0.13; 95% CI = 0.09–0.16) and a higher number of stressful life events (Adj. β = 2.62; 95% CI = 1.78–3.46), which were risk factors for depression in this sample. These results suggest that multifactorial discrimination is associated with increased exposure to multiple risk factors for poor mental health (Table 3).

**Table 3:**
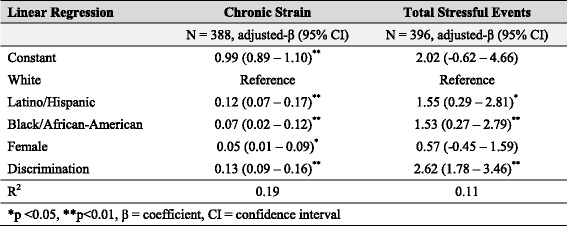
Effect of discrimination on risk factors for high depression scores

### Criterion 3: multifactorial discrimination influences protective factors for high depression

Multifactorial discrimination impacts individual-level, but not interpersonal-level, protective factors for high depression scores (Table 4). Individual-level protective factors were significantly inversely associated with discrimination. Discrimination was associated with reduced levels of mastery (Adj. β = −0.11; 95% CI = −0.16 – -0.06) and lower self-esteem (Adj. β = −0.27; 95% CI = −0.35 – -0.18).

**Table 4:**
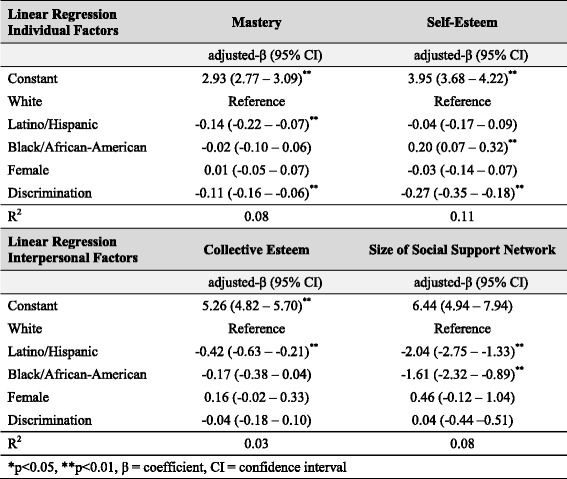
Effect of discrimination on protective factors for high depression scores (*N =* 396)

In the adjusted models, Latino/Hispanics had significantly lower mastery scores than Whites (Adj. β = −0.14; 95% CI = −0.22 – -0.07), and Black/African-Americans reported lower self-esteem than Whites (Adj. β = 0.20; 95% CI = 0.07–0.32). Overall, non-Whites had less access to individual-level resources that could help them cope with depression or other health challenges. Discrimination did not mediate this association, but acted as an independent predictor of protective psychosocial resources across all racial/ethnic groups.

However, discrimination was not a significant predictor of interpersonal protective factors. Although non-Whites had fewer people in their social support networks and lower collective esteem, these results were not affected by the addition of discrimination into the model, and discrimination was not associated with either social support network size or collective esteem (Table 4).

### Criterion 4: multifactorial discrimination affects health through alternate mechanisms

After adjusting for sex, race/ethnicity, chronic strain, and stressful life events, multifactorial discrimination remained a significant predictor of depression (Adj. OR = 1.68; 95% CI = 1.12–2.53; Table 5). Thus, the risk factors of chronic strain and stressful life events only partially mediate the effect of discrimination on depression, and discrimination increased the risk of depression through alternate, unmeasured mechanisms.

**Table 5:**
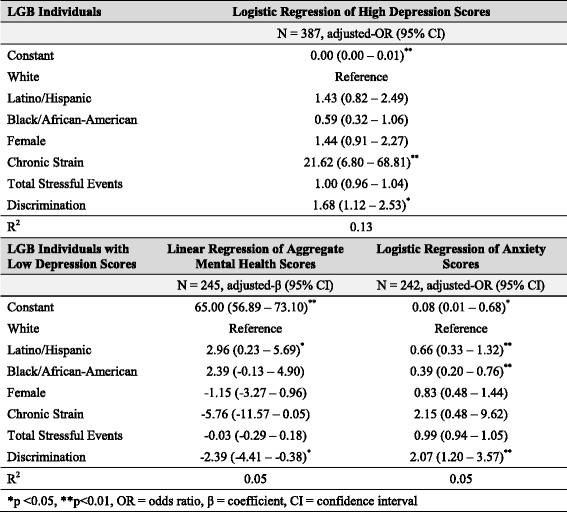
Effect of discrimination on depression and mental health outcomes after controlling for related risk factors

Discrimination also predicted mental health among individuals without depression. Controlling for risk factors (Table 5), discrimination remained a significant predictor of aggregate mental health scores (Adj. β = −2.39; −4.41 – -0.38) and anxiety (Adj. OR = 2.07; 1.20–3.57) among individuals with low depression scores. Among those with low depression scores, Latino/Hispanics had lower aggregate mental health scores (Adj. β = 2.96; 0.23–5.69), and both Latino/Hispanics (Adj. OR = 0.66; 0.33–1.32) and Black/African-Americans (Adj. OR = 0.39; 0.20–0.76) showed a higher risk of anxiety. The multifactorial discrimination these groups experience was negatively associated with overall mental health among individuals, even those without depressive symptoms, suggesting that discrimination can affect mental health through mechanisms other than depression or its risk factors.

### Multifactorial discrimination versus stigma

To determine if multifactorial discrimination was a fundamental cause of health inequities distinct from stigma, we repeated all analyses by first replacing discrimination with stigma in all models, and then by including both discrimination and stigma as covariates (data not shown). These sensitivity-analyses showed that while stigma was a significant covariate in the majority of the analyses, discrimination explained the outcomes of interest to a greater extent and more consistently than stigma.

When controlling for stigma, discrimination still met the criteria for a fundamental cause of health disparities. For example, adjusting for stigma, discrimination remained a significant predictor of depression (Adj. OR = 1.97; 95% CI: 1.28–3.02), while stigma was not a significant predictor (Adj. OR = 1.20; 95% CI: 0.84–1.70). In the linear regression models predicting total stressful life events and chronic strain, discrimination explained more of the variability in the outcome than did stigma, and discrimination remained significant in the fully adjusted models. Discrimination was significantly associated with mastery and self-esteem, even after adjusting for stigma. Finally, discrimination was positively associated with the odds of depression, even after controlling for other risk factors and stigma. In this study population, discrimination explained the variability in experiences of adversity more fully than stigma.

## Discussion

These results lend support to multifactorial discrimination as a fundamental cause of mental health inequities (Tables 2, 3, 4 and 5), given that discrimination meets all four criteria proposed by Link and Phelan [8]. To our knowledge, this is the first study to consider multifactorial discrimination as a fundamental cause of mental health inequities. Our results offer a possible explanation as to why multiple minority identities experience 1) multiple adverse psychological health outcomes, 2) higher exposure to risk factors, 3) less access to protective factors, and 4) poor mental health trajectories [14, 15, 34–36].

Our finding that multifactorial discrimination influences multiple psychological outcomes (Table 2) is consistent with other studies. Among Black/African-American LGBQ adolescents, racist and antigay discrimination was found to be associated with suicidal ideation and depressive symptoms [37]. Discrimination, including experiences of homophobia and racism, significantly predicted symptoms of psychological distress in a population of gay, Latino men [14]. Compared to White sexual minorities, more Black/African-American and Latino/Hispanic LGBQ subjects reported a history of serious suicide attempts [38]. Meanwhile, compared to heterosexual women of color and white sexual minority women, sexual minority women of color had greater risk of self-reported lifetime substance use [39].

Similarly, the results indicating that multifactorial discrimination affects multiple stress measures (Table 3) are substantiated by recent studies on multiple minority populations. One study showed that young Black/African-American men who have sex with men experience more distal stressors due to racism and homophobia [40]. Furthermore, in relation to advantaged groups, Black sexual minority women experience greater stress due to multifactorial discrimination on the basis of their triply disadvantaged social status in terms of sex, race, and sexual orientation [41, 42]. Research suggests that stress is a mediator of the relationship between discrimination and health in multiple minorities [14, 43, 44].

Moreover, the finding that multifactorial discrimination influences access to protective psychosocial resources is supported by studies that show lower levels of mastery and social support among diverse racial/ethnic LGBQs [45, 46], and studies that show these psychosocial resources buffer experiences of minority stress [6, 47, 48]. Interestingly, our results show that discrimination specifically affects access to individual-level protective factors such as mastery and self-esteem as opposed to interpersonal-level factors such as social support and collective esteem (Table 4).

Finally, our results highlight multifactorial discrimination as a continuous contributor to psychiatric morbidity even after accounting for stress-related risk factors (Table 5). These findings coincide with research examining the different pathways through which discrimination is capable of perpetuating health inequalities more broadly. While the alternative mechanisms through which discrimination affects mental health over time are unidentifiable in our analysis, research shows that multifactorial discrimination encountered by multiple minorities is associated with increased health-risk behaviors, which may be linked to various poor health outcomes [49–51]. Additionally, multifactorial discrimination may be responsible for widening health disparities by decreasing access to quality health care among marginalized communities. A nationwide survey found that non-White sexual minorities experienced higher rates of discrimination in health care settings relative to their White counterparts [52]. When compared to White sexual minorities, non-White sexual minorities were more likely to report fears and concerns about obtaining necessary health services due to past experiences of discrimination and substandard care [53]. In another study, African American sexual minority women who attributed their negative experiences in a healthcare setting to multifactorial discrimination decreased their health care utilization following the negative experience [54]. Among minority populations, discrimination during health care is significantly associated with delayed care and stopped treatment [55, 56]. Disengagement with the health care system due to negative patient-provider interactions, resulting from discrimination, may partially explain poor health outcomes across marginalized communities [57–59]. Given that discrimination can affect disease outcomes, through its effect on health care access, the relationship between discrimination and poor health is thought to be partly mediated by non-stress-related risk factors. Even if stress caused by multifactorial discrimination could be eliminated, research indicates that discrimination would continue to maintain and reproduce health inequalities through other intervening mechanisms.

Although multifactorial discrimination has not previously been identified as a fundamental cause of mental health inequities, recent research proposes stigma as a fundamental cause of health inequalities [33]. Hatzenbuehler, Link, and Phelan argue that stigma is an all-encompassing concept that includes various stigmatized statuses, including, but not limited to HIV status, obesity, sexual orientation, disability, and race/ethnicity. They also state that discrimination is a “constitutive feature of stigma” because “the overall stigma process incorporates several other elements, such as labeling and stereotyping”, making the concept broader than discrimination [33]. The *Project STRIDE* dataset did incorporate a stigma measure, which was moderately correlated with discrimination. However, we used the discrimination measure in our analysis not only for its validity and reliability [60], but also to address a specific theoretical framework that centers on discrimination as it relates to intersecting minority identities, considering individual, intersectional experiences and behavior. While stigma typically refers to beliefs and attitudes that lead people to fear, avoid, or reject those they perceive as different, discrimination is the behavioral manifestation of stigma [61]. Arguably, this quality makes discrimination easier to measure than stigma. Therefore, just as both racism and residential segregation have been established as fundamental causes despite being linked [9, 62], we believe our analysis provides strong evidence to include multifactorial discrimination as a fundamental cause in its own right.

### Limitations and future research directions

An important limitation concerning our analysis is that heterosexual Black/African-American and Latino/Hispanic individuals were not included in the *Project STRIDE* study sample. While we could not quantitatively distinguish between the adversity experienced by non-White LGBs and non-White heterosexuals, we were able to examine differences within sexual minorities. We structured our analysis such that we compared non-White LGBs to White LGBs, which allowed for an intersectional interpretation of discrimination among the multiple minorities, meaning that the discrimination experienced by non-White LGBs was related to their multiple, intersecting minority identities. Our analysis cannot prove that stress exposure, access to protective psychosocial resources, and the prevalence of mental health outcomes observed for non-White LGBs are any different than they would have been for Black/African-American and Latino/Hispanic heterosexuals. However, such differences are thoroughly supported in previous research [63, 64], and we are confident in the assumption that racial/ethnic sexual minorities experience additional and interacting burdens compared to heterosexual racial/ethnic minorities. Further research is needed to parse the association between multifactorial discrimination and mental health inequities in more diverse, heterogeneous populations. Such studies would allow for a better understanding of how social identities mutually interact with and simultaneously influence each other within varied contexts [65].

We were also limited in our evaluation of the fourth criterion of the fundamental cause theory, which speaks to the persisting nature of a fundamental cause in its ability to reproduce health inequalities through alternate, intervening mechanisms. Our source data included a 1-year follow-up of study participants, which is not long enough to demonstrate that discrimination causes health inequalities to persist over time. Notably, the fourth criterion of the fundamental cause theory describes a longitudinal, population-level phenomenon that cannot be effectively captured by the individual-level data from *Project STRIDE*. However, our limited analysis does establish discrimination as a significant predictor of depression after adjusting for relevant risk factors, and the association between discrimination and psychiatric morbidity endures even among those with low depression. This finding implicates unmeasured mechanisms through which discrimination reinforces and generates mental health inequities.

The inability to assess institutional discrimination was another limitation of our research. We were unable to analyze how institutions, such as the health care system, the education system, and the criminal justice system, may discriminate against multiple minorities, such as those in *Project STRIDE*. Since our measure of multifactorial discrimination only truly captures interpersonal discrimination, our understanding of multifactorial discrimination as a fundamental cause is limited to the interpersonal level in the context of this study. As a result, this analysis likely underestimates the pervasiveness of multifactorial discrimination as a fundamental cause of mental health inequities. Future research should consider institutional discrimination when exploring the effects of multifactorial discrimination on the health of multiple minorities.

Lastly, our focus on mental health inequities limited our ability to empirically test whether discrimination acts a fundamental cause of all health inequalities. However, other studies have shown that discrimination contributes to poor physical health outcomes [66–70]. Moreover, stress physiology research reveals that Black/African-American sexual minority males have a flatter diurnal cortisol curve compared to White sexual minority males [71], which has been extensively linked to poor mental health and physical health outcomes in the literature [72–76]. Future studies should incorporate physical health outcomes in their evaluation of multifactorial discrimination as a fundamental cause of health inequalities.

## Conclusions

In light of our findings, public health interventions would likely benefit from incorporating intersectional perspectives to adequately serve diverse populations. Currently, few epidemiological studies reflect intersectionality in their theoretical frameworks [77], and therefore, the evidence base informing intersectionality of intervention design and policy is sparse. Moreover, public health practitioners must continue to address the upstream, fundamental causes of inequalities as opposed to focusing on downstream risk factors alone.

While the fundamental cause theory is acknowledged in the public health field, much work remains to be done to implement effective interventions and policies to reduce the impact of fundamental causes on health disparities. Addressing multifactorial discrimination as a fundamental cause will likely involve collaborating across many disciplines, institutions, and fields to organize efforts aimed at eliminating disparities through community mobilization, advocacy, education, and policy change. As a fundamental cause of mental health inequities, multifactorial discrimination will continue to perpetuate health disparities until it is addressed at an upstream level.

## Abbreviations

LGB: Lesbian, gay, and bisexual
LGBQ: Lesbian, gay, bisexual, and queer
SES: Socioeconomic status
