# Erratum to: Multifactorial discrimination as a fundamental cause of mental health inequities

**DOI:** 10.1186/s12939-017-0557-3

**Published:** 2017-08-15

**Authors:** Mariam Khan, Misja Ilcisin, Katherine Saxton

**Affiliations:** 1 Public Health Program, Santa Clara University, 500 El Camino Real, Santa Clara, CA 95053 USA; 2 Department of Biology, Santa Clara University, 500 El Camino Real, Santa Clara, CA 95053 USA

## Erratum

Unfortunately, after publication of this article [1], it was noticed that the tables were not formatted correctly during the production process. The corrected tables can be seen here and the original article has been updated to reflect this.

**Table 1:**
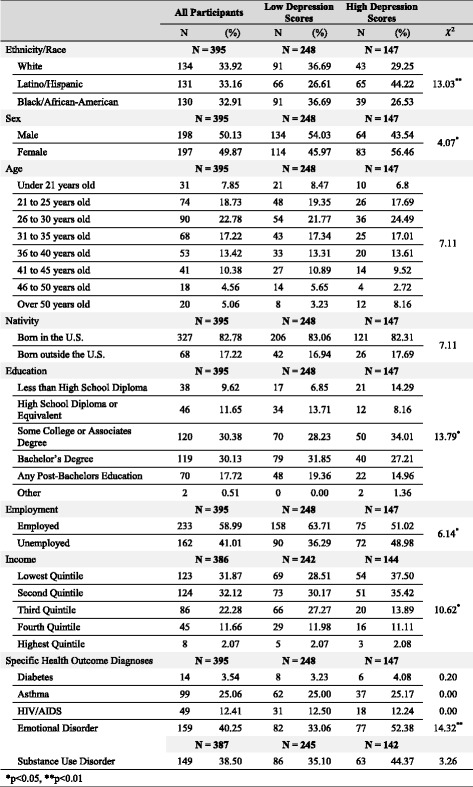
Demographics and health characteristics of LGB participants with high and low depression scores

**Table 2:**
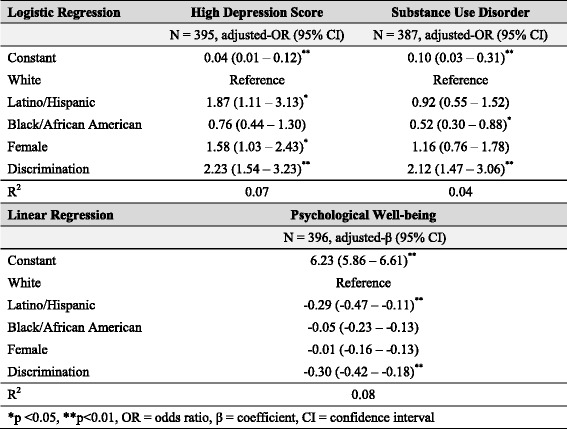
Effect of discrimination on health outcomes

**Table 3:**
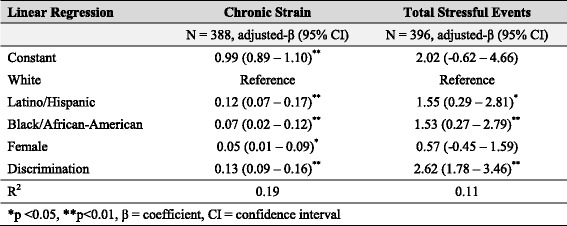
Effect of discrimination on risk factors for high depression scores

**Table 4:**
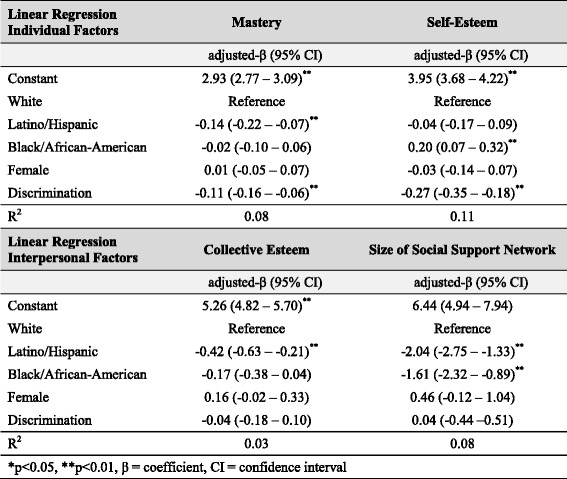
Effect of discrimination on protective factors for high depression scores (*N =* 396)

**Table 5:**
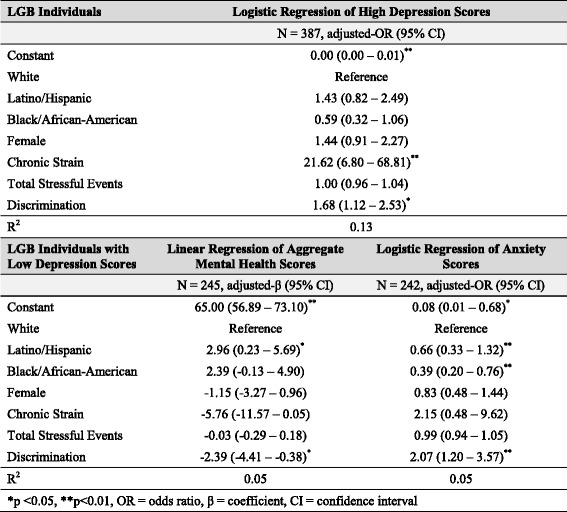
Effect of discrimination on depression and mental health outcomes after controlling for related risk factors
